# Fragment optimization for GPCRs by molecular dynamics free energy calculations: Probing druggable subpockets of the A_**2A**_ adenosine receptor binding site

**DOI:** 10.1038/s41598-017-04905-0

**Published:** 2017-07-25

**Authors:** Pierre Matricon, Anirudh Ranganathan, Eugene Warnick, Zhan-Guo Gao, Axel Rudling, Catia Lambertucci, Gabriella Marucci, Aitakin Ezzati, Mariama Jaiteh, Diego Dal Ben, Kenneth A. Jacobson, Jens Carlsson

**Affiliations:** 1 Science for Life Laboratory, Department of Cell and Molecular Biology, Uppsala University, BMC, Box 596, SE-75124 Uppsala, Sweden; 2 Science for Life Laboratory, Department of Biochemistry and Biophysics, Stockholm University, SE-10691 Stockholm, Sweden; 3 Molecular Recognition Section, Laboratory of Bioorganic Chemistry, National Institute of Diabetes and Digestive and Kidney Diseases, National Institutes of Health, Bethesda, Maryland 20892 United States; 4 Scuola di Scienze del Farmaco e dei Prodotti della Salute, Università degli Studi di Camerino, Via S. Agostino 1, 62032 Camerino (MC), Italy

## Abstract

Fragment-based lead discovery is becoming an increasingly popular strategy for drug discovery. Fragment screening identifies weakly binding compounds that require optimization to become high-affinity leads. As design of leads from fragments is challenging, reliable computational methods to guide optimization would be invaluable. We evaluated using molecular dynamics simulations and the free energy perturbation method (MD/FEP) in fragment optimization for the A_2A_ adenosine receptor, a pharmaceutically relevant G protein-coupled receptor. Optimization of fragments exploring two binding site subpockets was probed by calculating relative binding affinities for 23 adenine derivatives, resulting in strong agreement with experimental data (R^2^ = 0.78). The predictive power of MD/FEP was significantly better than that of an empirical scoring function. We also demonstrated the potential of the MD/FEP to assess multiple binding modes and to tailor the thermodynamic profile of ligands during optimization. Finally, MD/FEP was applied prospectively to optimize three nonpurine fragments, and predictions for 12 compounds were evaluated experimentally. The direction of the change in binding affinity was correctly predicted in a majority of the cases, and agreement with experiment could be improved with rigorous parameter derivation. The results suggest that MD/FEP will become a powerful tool in structure-driven optimization of fragments to lead candidates.

## Introduction

Fragment-based lead discovery (FBLD) has rapidly become a well-established technique in early drug development^1^. Several lead candidates developed using FBLD have already reached clinical trials, resulting in two FDA approved drugs^2^. In contrast to high-throughput screening (HTS), where large numbers (~10^5^–10^6^) of drug-sized molecules are tested experimentally, FBLD focuses on smaller libraries (typically 1000–5000 compounds) with molecules of low molecular weight (<300 Da)^2, 3^. By limiting the size of the molecules in the library, fragment screening achieves a much broader coverage of chemical space than HTS due to the astronomical number of possible drug-like molecules. The low molecular complexity of fragments also reduces the probability for steric mismatches with the receptor, leading to the discovery of ligands that optimally complement subpockets of the binding site^4, 5^. Consequently, screening of fragment libraries often delivers high hit-rates and diverse starting points for lead development^2, 3^. However, the ligands that emerge from fragment screening typically have low affinities and, in the second step of FBLD, these compounds need to be optimized to yield potent and selective lead candidates.

Fragment-to-lead optimization has proved to be a very challenging step in FBLD^2^. Prioritization of fragments for optimization is often guided by ligand efficiency (defined as the free energy of binding divided by the number of heavy atoms of the compounds^6^) and access to atomic resolution information regarding binding modes^7^. Recently, more intricate criteria, *e.g*. based on the thermodynamic binding profiles of the fragments, have also been suggested to be an important factor in ﻿t﻿he selection of starting points for optimization^8^. Two main strategies for fragment-to-lead optimization, “linking” and “growing”, have been proposed^2^. Although “linking” of compounds occupying different subpockets of a binding site has been successful in some cases, “growing” of the fragment by iterative additions of smaller chemical groups has become more widely used^2, 3^. In either case, access to high-resolution crystal structures of fragments bound to the target often makes crucial contributions to the optimization process^7^. Whereas computational methods for structure-based ligand design are routinely used for drug-sized molecules^9^, applications of such approaches to fragment optimization have been more scarce^10^. The fact that fragments are weak ligands, only occupy a small fraction of the binding site, and may have multiple binding modes adds extra levels of complexity that are challenging to predict with simplified models such as empirical scoring functions. Furthermore, scoring functions developed for computer-aided ligand design have been parameterized based on drug-like compounds, and it has been suggested that these may not be suitable for fragment ligands^11, 12^. Molecular dynamics (MD) simulations in combination with alchemical free energy methods, which explicitly consider contributions to binding from conformational flexibility and interactions with water molecules, could provide a rigorous approach to guide fragment optimization^13^, but this technique has only recently been applied to FBLD^10^. Accurate predictions of relative binding affinities for analogs to ligands identified by fragment screening could improve the efficiency of FBLD, further establishing this approach as a groundbreaking strategy for early drug development.

In this work, the utility of MD combined with alchemical free energy methods in fragment optimization was explored for the human A_2A_ adenosine receptor (A_2A_AR), a G protein-coupled receptor (GPCR) relevant for drug development^14^ against Parkinson’s disease^15^ and cancer^16^. Multiple high-resolution crystal structures of the A_2A_AR have recently been determined^17, 18^ and numerous fragment-sized ligands have been identified to this target^19–21^, making it an ideal test case for evaluating a computational approach. Calculation of relative binding affinities using MD simulations in combination with the free energy perturbation (MD/FEP) method was first benchmarked retrospectively for 23 fragment-sized compounds^22, 23^. The MD/FEP technique was also used to assess multiple binding modes and predict the thermodynamic signatures governing changes in binding affinity, which are both factors of major interest in the optimization process. In a second step, MD/FEP was applied prospectively to predict relative affinities for 12 fragment-sized compounds with unknown binding affinities, followed by experimental evaluation of these in pharmacological assays. In light of the results, the feasibility of using MD simulations in combination with alchemical free energy methods as a tool in fragment-to-lead optimization will be discussed.

## Results

### Mapping binding site subpockets using free energy calculations for fragment ligands

**Figure 1:**
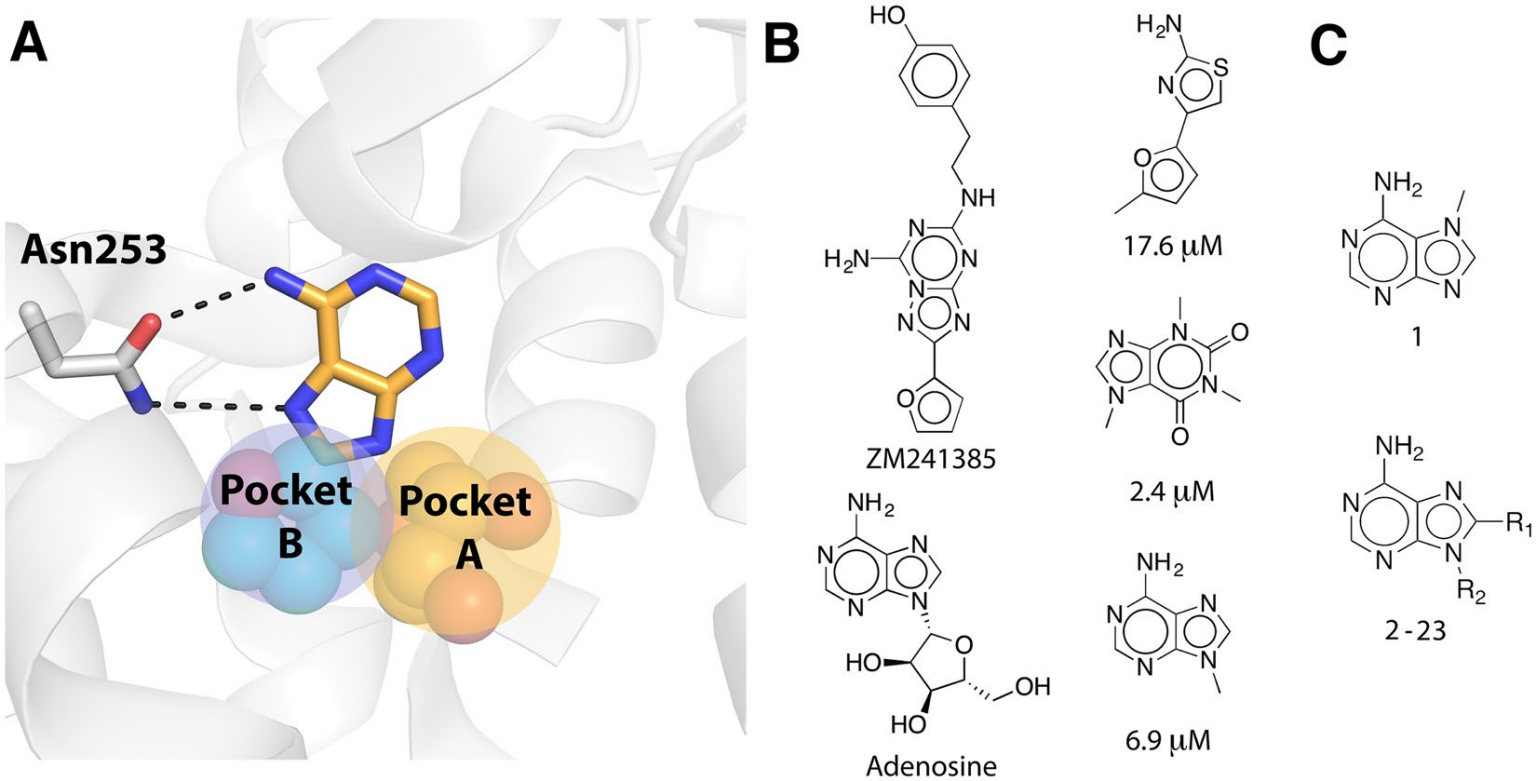
(**A**) Orthosteric binding site of the A_2A_AR shown as white cartoon with Asn253 in sticks. The adenine group is shown in sticks with carbon atoms in gold and hydrogen bonds indicated with black dashed lines. Two adjacent subpockets are shown as spheres with yellow (pocket A, ribose group of endogenous agonist adenosine from the crystal structure with PDB code 2YDO)^18^ and cyan (pocket B, furan group of antagonist ZM241385 from the crystal structure with PDB code 4EIY)^17^ carbon atoms. (**B**) Two adenine-based and three fragment-sized ligands of the A_2A_AR. K_i_ values are provided for the fragment ligands^21, 22, 24^. (**C**) 2D structures of compounds **1–23**. The R-groups are shown in Table 1.

**Figure 2:**
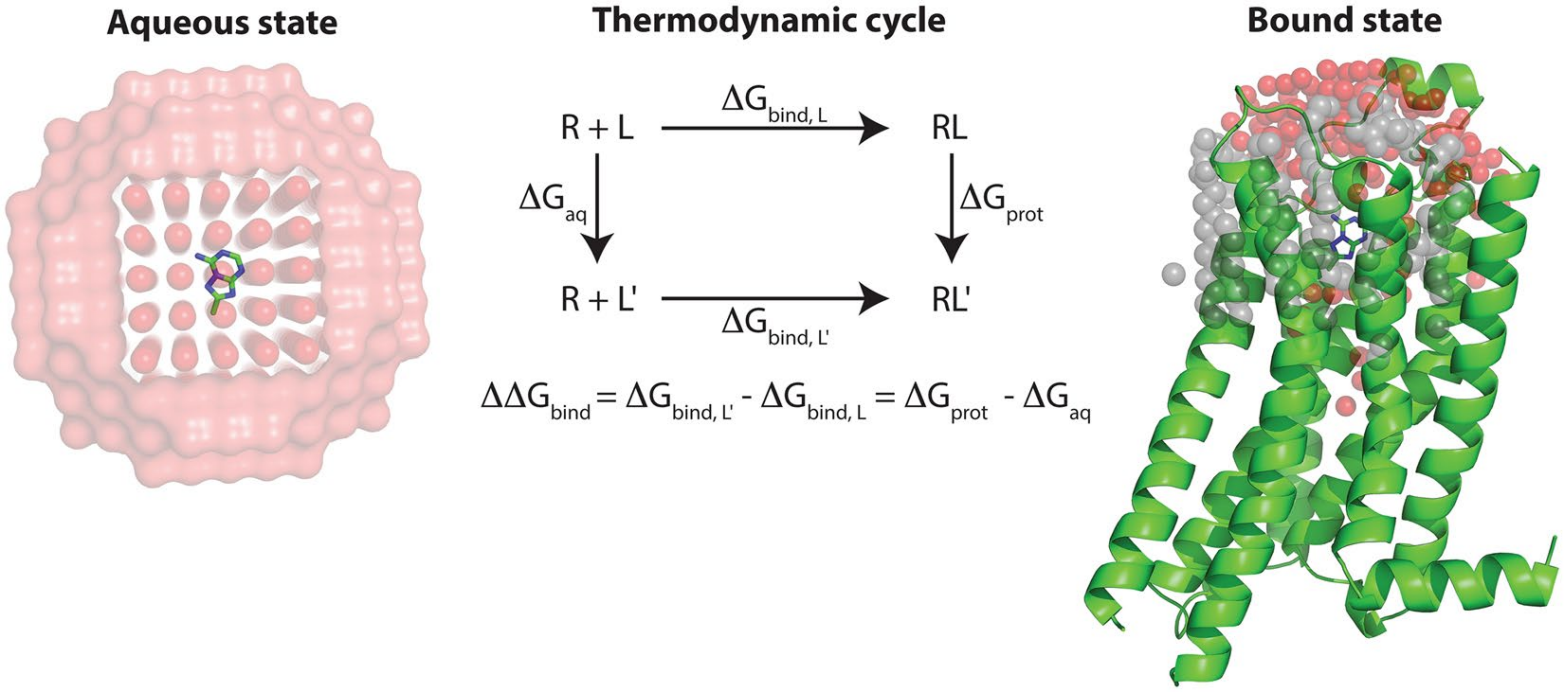
Thermodynamic cycle used to calculate relative free energies of binding (ΔΔG_bind_) from MD simulations. Alchemical transformations of the ligands L and L’ were performed in aqueous solution (ΔG_aq_, left panel) and bound to the receptor (ΔG_prot_, right panel). The protein is shown as green cartoon and the ligand is depicted in sticks. Water molecules are shown as red spheres and membrane carbon atoms are represented by grey spheres.

Analysis of available A_2A_AR crystal structures in complex with agonists^18^ and antagonists^17, 24^ revealed that the orthosteric site, *i.e*. the binding site of the native agonist, has several subpockets that could accommodate fragment-like ligands (Fig. 1). Hydrogen bonding to Asn253 has been identified as a key interaction for ligand recognition and this part of the binding site has also been demonstrated to be a hot-spot for fragment binding^21, 24^. Fragment-sized ligands occupying this region could be further optimized by extension into two additional buried subpockets of the orthosteric site (Fig. 1). The first of these is the ribose-recognizing site (pocket A) and the second is a pocket located below the adenine moiety of adenosine (pocket B). To explore if MD/FEP could guide fragment growth into the two different subpockets, relative binding free energies (ΔΔG_bind_) for 20 pairs of adenine-derived compounds (Table 1) were calculated using the thermodynamic cycle shown in Fig. 2. The relative binding affinity for a compound pair was calculated from alchemical transformations of one ligand into another in complex with the receptor and in aqueous solution (Fig. 2)^13^. Experimental binding affinities from radioligand binding assays were available for 20 adenine derivatives (**1**–**17**, **19**, **22**–**23**)^22, 23^ and were determined in this work for three additional adenine-based compounds (**18**, **20**, and **21**, Supplementary Table 1). The compound pairs differed by one to five heavy atoms and spanned up to >500-fold changes in binding affinity. Adenine-based ligands devoid of a ribose-like group are typically antagonists of the A_2A_AR, which was also confirmed for four selected compounds (**5**, **19**, **22**, **23**) by measuring inhibition of agonist-induced cAMP production (Supplementary Figure 1). Based on these results, a high-resolution structure of the A_2A_AR in an inactive conformation (PDB code 4EIY)^17^ was used in the simulations, and initial ligand binding modes were generated by aligning the adenine moiety to the adenine-like core of the co-crystallized antagonist. All MD simulations were performed in a spherical system centered on the binding site with explicit representation of protein, solvent, membrane, and ligand. Each MD/FEP calculation was divided into several steps, corresponding to transformations for electrostatics, Lennard-Jones, and relevant bonded force field energy terms. The number of steps and simulation length of each MD/FEP calculation were optimized to achieve convergence and the uncertainty of each step was <0.4 kcal/mol in all cases, with an average of 0.1 kcal/mol for all transformations. Three independent sets of simulations with an average total length of close to 100 ns were used to calculate the relative binding free energy for each compound pair (a total of 1.9 µs for the 20 compound pairs).

**Table 1:** Calculated and experimental relative binding free energies for 20 compound pairs based on adenine. 2D structures of the compounds are shown in Fig. 1C.

Ligand pair (A → B)		cmpd A		cmpd B		ΔΔG_bind_ (kcal/mol)	
		R_1_	R_2_	R_1_	R_2_	Experimental^a^	Calculated^b^
**1** → **2**	**pocket A**	—	—	H	CH_3_	<−1.6	−3.9 ± 0.2
**3 → 4**		Br	CH_3_	Br	H	2.0 ± 0.3	2.4 ± 0.0
**5 → 3**		Br	CH_3_CH_2_	Br	CH_3_	0.5 ± 0.3	−1.2 ± 0.0
**6 → 5**		Br	HOCH_2_CH_2_	Br	CH_3_CH_2_	−1.5 ± 0.2	−2.5 ± 0.1
**7 → 5**		Br	CH_3_CH_2_CH_2_	Br	CH_3_CH_2_	−1.1 ± 0.2	−0.5 ± 0.1
**8 → 5**		Br	(CH_3_)_2_CHCH_2_	Br	CH_3_CH_2_	−2.9 ± 0.3	−2.3 ± 0.1
**9 → 2**		H	CH_3_CH_2_	H	CH_3_	0.7 ± 0.2	0.2 ± 0.0
**10 → 9**		H	HOCH_2_CH_2_	H	CH_3_CH_2_	−1.0 ± 0.2	−1.4 ± 0.1
**11→ 9**		H	(CH_3_)_2_CHCH_2_	H	CH_3_CH_2_	<−2.3	−3.2 ± 0.0
**12 → 13**		H	HOCH_2_CH_2_CH_2_	H	CH_3_CH_2_CH_2_	0.5 ± 0.2	0.6 ± 0.4
**3 →2**	**Pocket B**	Br	CH_3_	H	CH_3_	2.4 ± 0.2	2.3 ± 0.2
**5 → 9**		Br	CH_3_CH_2_	H	CH_3_CH_2_	2.2 ± 0.3	1.5 ± 0.0
**14 → 15**		Br	cC_5_H_9_	H	cC_5_H_9_	0.0 ± 0.3	−2.2 ± 0.2
**16 → 17**		Br	CH_2_CHCH_2_CH_2_	H	CH_2_CHCH_2_CH_2_	1.0 ± 0.2	0.3 ± 0.2
**18 → 9**		CH_3_	CH_3_CH_2_	H	CH_3_CH_2_	1.4 ± 0.2	1.4 ± 0.0
**19 → 9**		furyl	CH_3_CH_2_	H	CH_3_CH_2_	3.8 ± 0.2	5.4 ± 0.2
**20 → 9**		OH	CH_3_CH_2_	H	CH_3_CH_2_	0.4 ± 0.2	0.1 ± 0.1
**21 → 18**		CH_3_O	CH_3_CH_2_	CH_3_	CH_3_CH_2_	1.3 ± 0.1	2.7 ± 0.1
**22 → 21**		CH_3_CH_2_O	CH_3_CH_2_	CH_3_O	CH_3_CH_2_	−0.3 ± 0.2	−0.4 ± 0.0
**23 → 21**		(CH_3_)_2_CHO	CH_3_CH_2_	CH_3_O	CH_3_CH_2_	<−4.9/−0.8^c^	0.0 ± 0.1

^a^Uncertainties are calculated as the standard error of the mean based on the maximal and minimal affinities values obtained from the 95% confidence intervals of the experimentally determined K_i_ values. Experimental K_i_ values can be found in Supplementary Table 1.

^b^Average relative binding free energy from three independent trajectories with uncertainties estimated as the standard error of the mean.

^c^K_i_ value from reference 23/Remeasured K_i_ value in this work (Supplementary Table 1).

**Figure 3:**
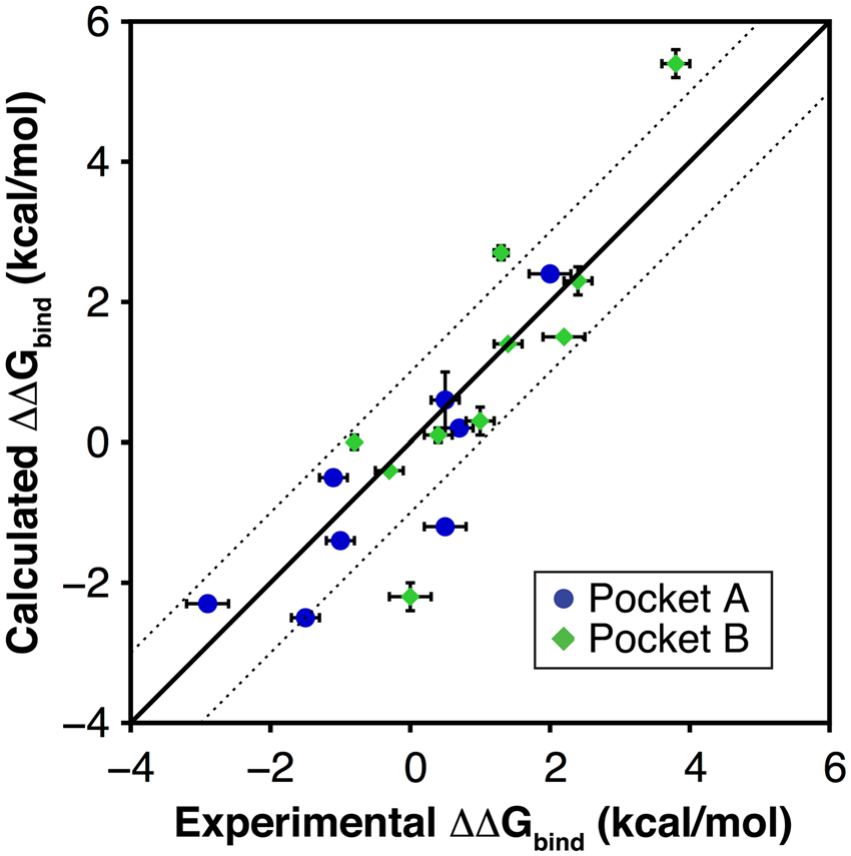
Comparison of calculated and experimental relative binding free energies (ΔΔG_bind_) for 18 compound pairs. The solid line represents prefect agreement between calculated and experimental data whereas the dotted lines represent an absolute deviation of 1 kcal/mol. Experimental and computational error bars correspond to the data reported in Table 1.

The first set of 10 pairs of adenine derivatives (Table 1) probed opportunities for growing fragments into the ribose-recognizing site (pocket A, Fig. 1) and mainly involved substitutions in the N9-position of the adenine scaffold (Table 1). The average unsigned error for this set of relative binding free energies was 0.66 kcal/mol, resulting in strong correlation with experimental binding data (Fig. 3, R^2^ = 0.75). Relative free energies involving compounds **1** and **11** were not included in the analysis of correlation with experimental data as reliable K_i_ values could not be determined for these compounds due to their lack of binding at the highest tested concentration (K_i_ > 100 μM). However, it should be noted that MD/FEP correctly predicted the direction of the shift in binding free energies in both cases. One example of successful fragment growth into pocket A was observed for addition of a methyl group in the N9-position of the adenine scaffold (compounds **3** and **4**). This resulted in a large improvement of the binding affinity (2.0 kcal/mol), which was also reproduced by the calculated free energy change of 2.4 kcal/mol. Interestingly, substituents larger than two heavy atoms in pocket A typically reduced binding affinities. For example, replacement of a 9-ethyl substituent by 2-hydroxyethyl, isobutyl or propyl moieties led to losses of binding affinity, which were also captured the MD/FEP calculations.

The second compound set explored the effects of substituents in the C8-position of the adenine scaffold, which extended into pocket B. A bromine in this position (compound **3**) led to a 58-fold increase in affinity compared to the unsubstituted compound **2**. This effect was also captured by the free energy calculations, which predicted an improvement in binding corresponding to 2.3 kcal/mol between compounds **2** and **3**, in close agreement with the experimental value (2.4 kcal/mol). Interestingly, changes in affinity from addition of a bromine in the C8-position were dependent on the substituent in the N9-position. This interdependency of the two substituents was also captured by the calculated binding free energies for this subset of four compound pairs (**2**–**3**, **5**, **9**, and **14**–**17**). Another series of compounds with an ethyl group in the N9-position and varying substituents in C8-position (**9** and **18**–**23**) was also considered. A 10-fold increase of binding affinity compared to compound **9** was obtained for the C8-methyl substituent (compound **18**) and addition of a hydroxyl group in the same position (compound **20**) also resulted in improved binding. The relative binding free energies for a majority of the considered pairs exploring pocket B were within 1 kcal/mol of the experimental value (Fig. 3). However, for the pair consisting of 8-alkoxy-9-methyladenine derivatives **23** and **21**, there was a large discrepancy between the published experimental affinity and relative binding free energy calculated from MD simulations. The experimentally determined values^23^ indicated a >1000-fold loss of binding affinity, as compound **23** showed no significant binding (reported K_i_ value > 100 μM), whereas the calculated value suggested that the two compounds had similar affinity. As this was a major outlier among the considered compound pairs, compound **23** was retested in a radioligand binding assay at the A_2A_AR. The K_i_ value was determined to be 95 nM for compound **23** (Supplementary Table S1), leading to a relative free energy of −0.8 kcal/mol, which was in better agreement with the prediction and resulted in a strong correlation with experiment for the second compound set (R^2^ = 0.75, Fig. 3).

The ability of MD/FEP to predict changes in affinity was further highlighted by the strong correlation between experimental and predicted binding free energies for the full set of 18 compound pairs (R^2^ = 0.78, Fig. 3). To assess the influence of experimental uncertainties on this result, the correlation was calculated for 1000 random selections of either the maximal or minimal K_i_ value obtained from the 95% confidence interval, which resulted in R^2^ = 0.75 with a standard deviation of 0.1. It should also be noted that the correlations between the experimentally determined relative binding affinities and trivial size-descriptors, *e.g*. the difference in the number of heavy atoms (R^2^ = 0.10) or predicted 1-octanol/water partition coefficients (AlogP, R^2^ = 0.44), were low. In order to compare our results to an empirical scoring function, the adenine derivatives were also docked to the orthosteric site using the GLIDE docking program^25^ and binding free energies were calculated with the standard precision (SP) scoring function for the 18 compound pairs. All of the docked compounds reproduced the binding mode expected from crystal structures of the A_2A_AR in complex with adenine-based ligands. The correlation with experimentally determined relative binding free energies for GLIDE-SP (R^2^ = 0.42, Supplementary Figure 2) was similar to that obtained for ALogP and significantly lower than for MD/FEP.

### Assessment of alternative binding modes and thermodynamic signatures of fragment binding

**Figure 4:**
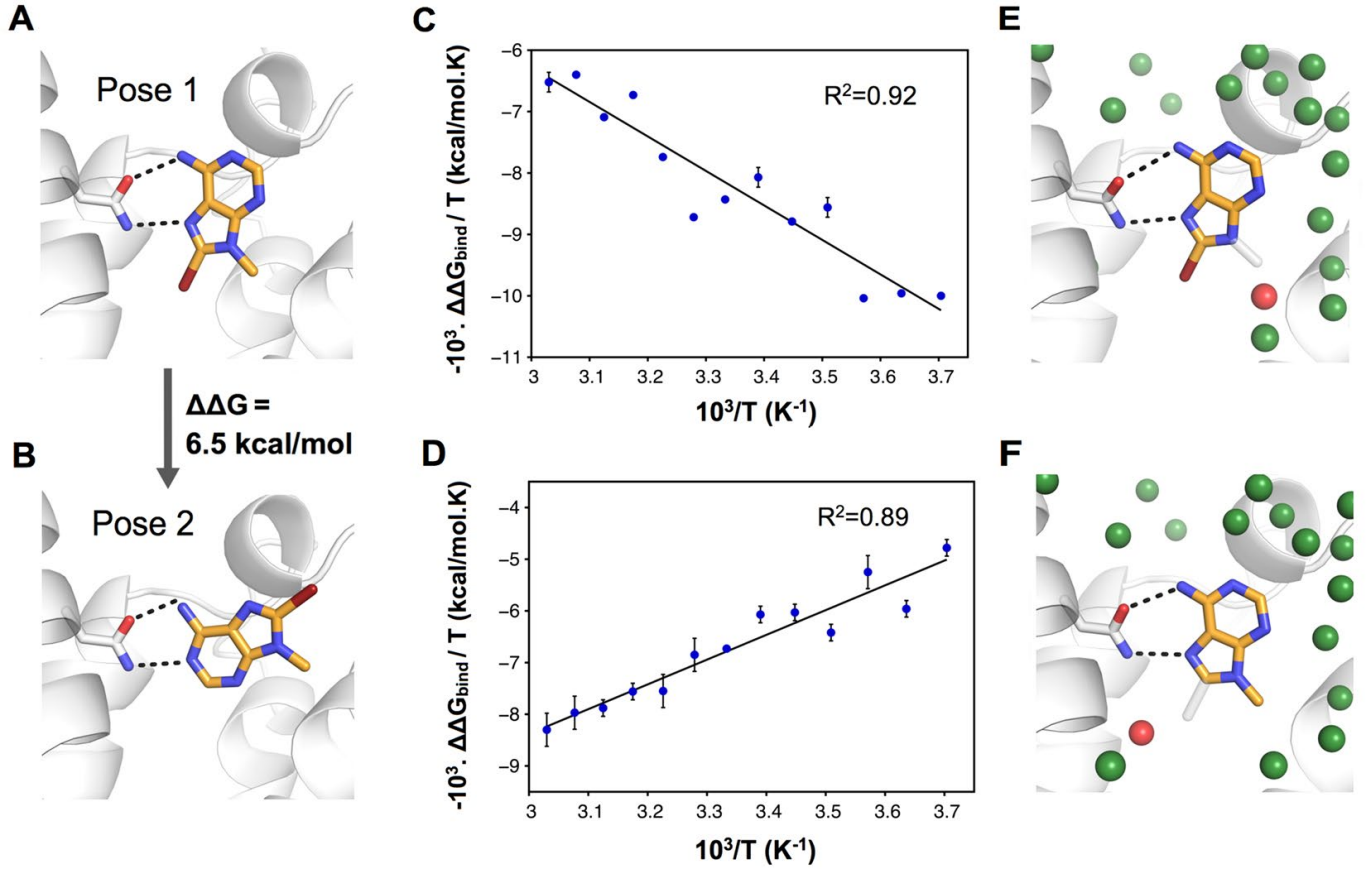
(**A**) Binding mode of compound **3** based on an A_2A_AR crystal structure in complex with a related ligand (PDB code 4EIY). (**B**) Alternative binding mode for compound **3**. (**C,D**) Determination of entropy and enthalpy components of the relative binding free energy from MD/FEP calculations at different temperatures for compounds **3** and **4** (**C**), and compounds **2** and **3** (**D**). (**E,F**) Maps of binding site solvent structure from clustering of snapshots from a simulation of compound **4** (**E**) and **2** (**F**) in complex with the A_2A_AR. The corresponding bromine and methyl substituents in compound **3** are represented as transparent grey sticks. In both cases, the water molecule displaced by compound **3** is shown as a red sphere. The orthosteric binding site of the A_2A_AR is shown as a white cartoon with a key residue in sticks. The binding modes of the ligands are shown in sticks with carbon atoms in gold and hydrogen bonds indicated with black dashed lines.

The use of MD/FEP in fragment optimization could be limited by the uncertainty associated with ligand binding modes if a crystal structure of the complex is not available. As fragments are small, such compounds can bind in a large number of orientations and it may be challenging to rank these with more simplified models, *e.g*. molecular docking scoring functions^12, 26^. The calculations for 9-methyl adenine derivatives **2** and **3** were extended to explore two alternative binding modes identified by the molecular docking study carried out by Lambertucci *et al*.^22^ (Fig. 4A and B). Both proposed binding modes predicted a hydrogen bond between the exocyclic nitrogen of the adenine moiety and the side chain oxygen of Asn253. The first binding mode involved an additional hydrogen bond between the N7 of the adenine-moiety and the side chain nitrogen donor of Asn253, leading to an orientation that was essentially identical to that observed in the crystal structure of the A_2A_AR in complex with adenosine (Fig. 1)^18^. In the alternative orientation, a hydrogen bond with the N1 atom of the adenine moiety was instead obtained, resulting in a second distinct binding mode. The relative binding affinity of the two poses (Fig. 4A and B) was calculated by alchemically transforming one binding mode into the other via an intermediate compound (**24**) to assess their probability (Supplementary Figure 3). The calculated free energies suggested that the pose derived from the binding mode of adenosine in A_2A_AR crystal structures, which was also used in the MD/FEP calculations, was favored by 6.5 ± 0.1 kcal/mol. The population of the alternative binding mode was thus predicted to be very low and would not influence the experimentally measured binding affinity significantly.

The enthalpic and entropic components of the binding free energy are increasingly attracting interest in drug discovery as these can provide more information on the driving forces of ligand binding^27^. Although the experimental binding free energy differences for compound **3** relative to compounds **2** and **4** were accurately reproduced by the MD/FEP calculations, it was not clear from visual inspection why the addition of a single heavy atom resulted in such a large change in binding affinity in both cases. To further quantify the change in binding free energy, it was decomposed into enthalpy and entropy components using a relationship analogous to the van’t Hoff equation. MD/FEP calculations were carried out for the two compound pairs at 13 different temperatures between 270 and 330 K. The enthalpy and entropy components could then be derived from the slope and intercept of the relation between ΔΔG_bind_/T and 1/T (Fig. 4C and D)^28^. These calculations demonstrated that the predicted affinity increase for compound **3** relative to compound **2** was driven by entropy (−TΔΔS_bind_ = −7.1 kcal/mol), which was counteracted by an unfavorable enthalpy contribution (ΔΔH_bind_) of  +4.8 kcal/mol. In contrast, the gain in affinity for compound **3** relative to compound **4** was enthalpy driven (ΔΔH_bind_ = −5.6 kcal/mol and −TΔΔS_bind_ =  + 3.4 kcal/mol). Overall, there were only small differences in receptor structure between the three complexes based on the MD simulations, suggesting that changes in the solvent network could be responsib﻿le for the distinct thermodynamic profiles. MD snapshots of the water molecules in the binding site were clustered to identify hydration sites in the vicinity of the ligands using the algorithm developed by Young *et al*.^29^. Comparison of the solvent networks revealed that introduction of the 9-methyl group (compound **2**) displaced an ordered water molecule in pocket A (Fig. 4E) whereas the 8-bromine (compound **3**) replaced a different ordered water in pocket B (Fig. 4F). The large increases in binding affinity hence appeared to be connected to changes in binding site solvation in both cases, but were the result of different thermodynamic profiles.

### Prospective predictions for three nonpurine fragment series

**Figure 5:**
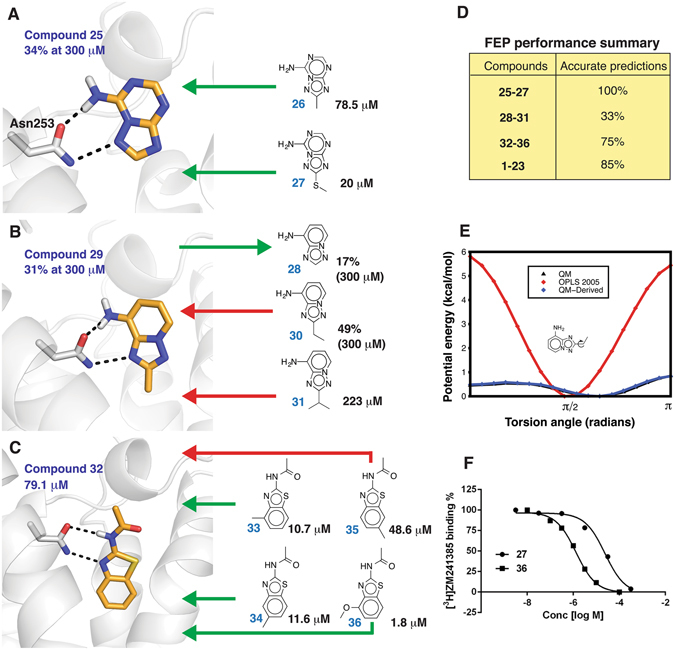
(**A**–**C**) Binding modes and summary of MD/FEP predictions for three nonpurine compound series. The orthosteric binding site of the A_2A_AR is shown as a white cartoon with key residues in sticks. The predicted binding modes of the ligands are shown in sticks with carbon atoms in gold and hydrogen bonds indicated with black dashed lines. The experimental result for each fragment is shown as its K_i_ (μM) or % displacement of radioligand binding at 300 μM. The performed MD/FEP calculations are represented with arrows in red and green, which corresponds to accurate and erroneous predictions, respectively. (**D**) Summary of agreement of MD/FEP calculations with experimental data. The percentage of accurate predictions of the direction of the binding free energy change is shown. (**E**) Potential energy curve for the indicated torsion calculated from OPLSAA_2005, DFT (QM), and a molecular mechanics potential fitted to the DFT results (QM-Derived). (**F**) Concentration-effect curves for displacement of radiolabeled A_2A_AR antagonist [^3^H]ZM241385 by compounds **27** and **36**.

To further challenge the MD/FEP method, calculations were extended to 12 fragment-sized nonpurine heterocycles of unknown affinity, which represented three series with varying levels of modeling difficulty (Fig. 5). The first two compound series were adenine-like and had substituents that explored pocket B. Compound **25**, [1,2,4]triazolo[1,5-*a*][1,3,5]triazin-7-amine, was a substructure of the A_2A_AR antagonist ZM241385 (Fig. 1). Similar to the adenine derivatives described in previous sections, this scaffold represented a case with high confidence regarding the fragment binding mode and predictions were made for three compounds in this series (**25**–**27**). The second scaffold, derivatives of [1,2,4]triazolo[1,5-*a*]pyridin-8-amine **28**, was less similar to adenine, but was assumed to maintain hydrogen bond interactions with Asn253 (Fig. 5), and relative affinities were predicted for three 2-alkyl analogs (**29–31**) that probed pocket B. The third series of five variously substituted derivatives of *N*-(benzo[*d*]thiazol-2-yl)acetamide **32** was unrelated to adenine. This represented the most challenging series, as the binding mode of this scaffold was unknown. After the MD/FEP predictions had been completed, the 12 compounds were evaluated experimentally using radioligand binding assays, and K_i_ values were determined for the ligands that showed >50% displacement at 300 μM (Supplementary Table S2). The computational and experimental results for the three series of fragments are summarized in Fig. 5.

The relative binding affinities for substituted triazolo-triazin-amine derivatives **26** and **27** compared to unsubstituted **25** were calculated using the same protocol as for the series of adenine derivatives. The MD/FEP calculations predicted that both compound **26** (0.6 kcal/mol) and **27** (4.3 kcal/mol) had higher affinity than compound **25**, which was also confirmed experimentally. Compounds **26** and **27** had K_i_ values of 78.5 and 20 μM, respectively, which were large improvements over compound **25** that only displayed 34% radioligand displacement at 300 μM. For the second series of analogs, the calculated relative binding free energy (ΔΔG_calc_ = 0.4 kcal/mol) correctly predicted that the 2-methyl substituted triazolopyridine **29** (31% at 300 μM) had higher affinity than compound **28**, which showed close to negligible radioligand displacement at 300 μM (17%). However, the 2-ethyl and 2-isopropyl substituted compounds **30** (49% at 300 μM) and **31** (223 μM) were incorrectly ranked relative to compound **29** (31% at 300 μM). Both compounds **30** and **31** were predicted to be weaker than compound **29** by 0.9 and 2.2 kcal/mol, respectively. The discrepancies for triazolopyridines **30** and **31** were intriguing considering the close agreement with experiment for the adenine-like ligands. To understand the origin of these erroneous predictions, the MD simulation trajectories for the ethyl-substituted compound **30** and methoxy-substituted compound **21** were inspected visually. The main difference between compounds **30** and **21** was found to be the torsional angles of the substituents protruding into pocket B. For compound **21**, the methoxy group primarily sampled angles that were within the plane of the adenine moiety, whereas the ethyl group did not align with the plane of the aromatic ring. Torsion angle scans for compound **30** using density functional theory (DFT) revealed large errors in the force field parameters both in the location of the minimum and the energy barrier height of the potential energy curve (Fig. 5E), whereas there was reasonable agreement between OPLSAA_2005 and DFT for compound **21** (Supplementary Figure 4). MD/FEP calculations were then repeated for compounds **30** and **31** using a force field term for the torsion that reproduced the DFT calculations. The calculated relative binding free energies (to compound **29**) changed from −0.9 to  + 0.1 kcal/mol for compound **30** and from −2.2 to + 0.7 kcal/mol for compound **31**. These shifts in calculated values resulted in accurate ranking of the two ligands relative to **29** (Supplementary Table S2).

For the third series of compounds, 2-acetamido-benzothiazole (compound **32**) was the core scaffold and had a K_i_ value of 79 μM. 4-Hydroxy-*N*-(4-methoxy-7-morpholinobenzo[*d*]thiazol-2-yl)-4-methylpiperidine-1-carboxamide (tozadenant), a compound that has been in clinical trials for the treatment of Parkinson’s disease^30^, could essentially be considered as a superstructure of this fragment. However, tozadenant could not be accommodated in the crystal structure used for the adenine derivatives in a manner that allowed for hydrogen bonding with residue Asn253. Hence, prior to experimental evaluation of this fragment series, an alternative binding site conformation based on a different A_2A_AR crystal structure^24^ was used. In this conformation, alternative side chain rotamers for His264 and Glu169 lead to a more open binding pocket, which could accommodate tozadenant and compound **32**. This binding pose for compound **32** was found to be stable in MD simulations, and a representative snapshot was used as starting point for the FEP calculations (Fig. 5C). The effects of adding a methyl group at three different positions (4, 5 and 6) of the benzothiazole ring of compound **32** were evaluated computationally (Fig. 5C, compounds **33**–**35**). Improvements of affinity corresponding to 0.3 and 0.4 kcal/mol were predicted by MD/FEP for compounds **33** and **34** respectively, whereas a large loss of binding was obtained for compound **35**. The predictions for compounds **33** and **34** agreed reasonably well with the subsequently determined 7-fold increases of affinity. Compound **35** was the weakest ligand of the three analogs with only a two-fold increase of affinity, but the MD/FEP calculations had predicted a loss of binding free energy in this case (2.7 kcal/mol). To further optimize compound **33**, MD/FEP calculations were carried out for the 4-methoxy-substituted compound **36**. The experimentally determined 44-fold increase of affinity (corresponding to 2.3 kcal/mol, Fig. 5F) compared to compound **32** was partially captured by the MD/FEP calculations, which predicted a 0.8 kcal/mol improvement in binding free energy. Hence, whereas the direction of the change in binding affinity was correct, the magnitude of the improvement in affinity was underestimated. To investigate if prediction accuracy could be improved by increasing sampling, we retrospectively extended the simulations by doubling the production time for the transformation between compounds **36** and **32**, which resulted in improved agreement with experimental data (ΔΔG_bind_ = 1.3 kcal/mol).

## Discussion

The focus of this work was to evaluate using MD simulations in combination with free energy calculations as a tool for fragment optimization. Three key results emerged from calculations of relative binding affinities for fragments ligands of the A_2A_AR, a GPCR that has been intensively studied as a drug target^14^. First, there was a strong correlation between calculated and experimental relative binding free energies for ligands based on an adenine scaffold. Remarkably, the direction of the shift in binding free energy was correctly predicted for all of the adenine derivatives that had an absolute experimental free energy change >0.5 kcal/mol. Second, the potential of MD/FEP calculations to assess alternative binding modes and predict thermodynamic signatures of fragment binding was demonstrated, which could be used to tailor ligand properties during optimization. Finally, prospective predictions for three compound series and evaluation of these in pharmacological assays highlighted opportunities and challenges for the use of MD/FEP calculations in FBLD.

The potential of MD/FEP to guide fragment optimization was clearly demonstrated by the excellent results obtained for the series of adenine derivatives^22, 23^. Substantial changes in affinity could be achieved by introduction of substituents in the C8- and N9-positions of this scaffold. These effects were not obvious by visual inspection of the complexes and changes in affinity did not correlate with trivial descriptors such as heavy atom count. The fact that addition of diverse substituents to the same subpocket improved binding affinities likely reflects a complex interaction network involving structural water molecules, polar and non-polar side chains. These effects were accurately captured by the MD/FEP calculations, but not by docking in combination with an empirical scoring function. As previously demonstrated by Warren *et al*., empirical scoring functions are more suitable for screening of large chemical databases to prioritize compounds for experimental testing rather than ranking closely related ligands by affinity^31^. The improved accuracy for MD/FEP may be due to explicitly taking into account water molecules, induced fit effects and associated entropic contributions to the binding^9^. The performance of docking scoring functions can also be further improved for specific targets by considering the effects of specific water molecules^22, 32, 33^, but such protocols may not be readily transferred to other ligand series or targets. Consideration of the enthalpic and entropic components of the binding free energy has recently been suggested to be an important metric to guide fragment optimization^8^. In addition to improved predictions of relative affinities, MD/FEP calculations make it possible to characterize the driving forces behind a change in free energy, which has previously been applied successfully to study ion hydration^28^ and enzyme catalysis^34^. In this work, we used the same approach to investigate the large differences in binding observed for two compound pairs from the series of adenine-based ligands. The affinity gains obtained for a substituent in the C8-position of adenine were found to be associated with a large increase of entropy and displacement of a binding site water molecule in pocket B, which appeared to be a classic example of the hydrophobic effect. Interestingly, the same hotspot has previously been identified based on MD-derived maps of the solvent network in the A_2A_AR binding site^35, 36^. In contrast, the addition of methyl substituent in the N9-position, which involved displacement of a water molecule from pocket B, led to a decrease of entropy and the improvement of the binding affinity was instead driven by enthalpy. Large improvements of affinity due to the addition of a single heavy atom, which has been referred to as the “magic methyl” effect, may hence have completely different thermodynamic origins. Although the predictions of the entropy and enthalpy contributions to the relative binding free energies will need to be further tested by comparison to experimental data, our results suggest that MD/FEP is not limited to guiding affinity optimization, but can also be used to tailor the thermodynamic profile of ligands.

Application of the MD/FEP technique to fragment-sized molecules has several advantages from a methodological standpoint. Molecular mechanics force fields are likely more accurate for fragment- than for drug-like compounds as parameters are typically developed based on fragment-sized molecules^37^. Furthermore, it should be more feasible to reach convergence of the free energy calculations for fragments as such molecules typically have fewer degrees of freedom than drugs. Comparison of our results to a recent study that applied the MD/FEP technique to two series of lead-like A_2A_AR ligands supports this idea^38^. The lower correlation with experiment obtained for two series of adenine-derived ligands may reflect that these were of lead-like size and interacted with the flexible extracellular loops whereas the fragments considered in this work had limited conformational flexibility and extended into a relatively rigid TM region. Interestingly, two recent benchmarks of binding free energy calculations for a large number of soluble targets showed a similar trend^10, 39^. It should be noted that access to information regarding the binding mode for a representative ligand was likely a key contributor to the accuracy of the MD/FEP calculations in all cases. Hence, if a high-resolution structure of a representative complex is available, MD/FEP calculations can be a valuable technique for ligand optimization and the approach appears to be particularly suited for fragments.

The prospective predictions carried out for three different fragment series revealed potential pitfalls for the use of MD/FEP to predict ligand binding affinities. In this case, the compounds had affinities in the high micromolar to millimolar range, which closely mimicked the scenario encountered in fragment-to-lead optimization^3^. In agreement with the results obtained for the adenine derivatives, the predictions were excellent for the fragments derived from a ligand co-crystallized with the A_2A_AR. The second fragment series illustrated the importance of high quality torsional force field parameters for small molecule ligands. The MD/FEP calculations based on OPLSAA_2005 parameters were not in agreement with experimental data, whereas a DFT*-*derived torsional potential resulted in correct ranking of the compounds. This result suggests that force field parameters should be used with caution even for fragment-sized molecules. As we focused only on congeneric series of compounds with mainly non-polar substituents, the performance of the partial atomic charges from OPLSAA_2005 was not assessed in this work. For example, consideration of different heterocyclic compounds could involve large changes in charge distribution, which may not be accurately represented by empirical partial charges. Fortunately, as fragments will have a small number of atoms and rotatable bonds, torsions and partial charges could in principle be derived using *ab initio* methods prior to the MD/FEP calculations to further enhance modeling accuracy. In line with these ideas, particular focus was put on improvements of torsional potentials and partial atomic charges in the recently released OPLS3 force field for organic molecules^40^. The last series of fragments illustrated challenges associated with lack of crystal structure information regarding fragment binding modes. Modeling of the binding mode involved consideration of several binding site conformations and required expert knowledge regarding ligand recognition by the target. Encouragingly, the direction of the change in binding free energy was correctly predicted in three out of four cases, but the magnitude of the shift in affinity was not always captured. Considering the many uncertainties involved in modeling of fragment binding modes, fragment optimization in the absence of a crystal structure should be considered to be very challenging. In these instances, the use of metadynamics^41^ and MD/FEP in combination with mutagenesis studies^42, 43^ to identify ligand binding modes hold promise. Binding modes that reproduce the initial structure-activity relationships could be used to guide compound selection in the following rounds of optimization.

The major advances made in molecular and structural biology for GPCRs^44, 45^ make it possible to apply FBLD to numerous targets of therapeutic interest. In the case of the A_2A_AR, fragment screening against stabilized receptor constructs by biophysical methods^19, 20^ and computationally using molecular docking^21^ have led to the discovery of diverse starting points for development of lead compounds. The determination of multiple high-resolution crystal structures of GPCRs in complex with fragments^24, 46^ provides exciting opportunities to apply computational methods in FBLD for GPCRs. Our results demonstrate that the MD/FEP approach can contribute to efficient optimization of fragment hits, which is key for successful use of FBLD in drug development. The combination of molecular docking screening for fragment identification and efficient ligand optimization via MD/FEP has the potential to become a powerful addition to the toolbox of methods used in fragment-based drug discovery.

## Methods

### MD/FEP calculations

The MD simulations were performed using a high-resolution crystal structure of the A_2A_AR (PDB accession code: 4EIY, 1.8 Å)^17^. In a first step, a hydrated 1-palmitoyl-2-oleoyl phosphatidylcholine (POPC) membrane bilayer was first equilibrated around the A_2A_AR structure with periodic boundary conditions using the 4.5.5 version of GROMACS^47^. These simulations were setup using the GPCR-ModSim protocol^48^ and the OPLS all atom (OPLSAA) force field^37^, TIP3P waters^49^, and Berger lipid parameters^50^. All protein atoms were tightly restrained to their initial coordinates and the hydrated membrane was equilibrated for a total of 40 ns at 300 K. All MD/FEP calculations were carried out starting from the membrane equilibrated A_2A_AR system using the program Q^51^ with the same force field. Ligand parameters were obtained using the OPLSAA_2005 version implemented in the program hetgrp_ffgen (Schrödinger, LLC, New York, NY, 2017). The simulations were carried out at 310 K in a sphere of 18 Å radius centered on the ligand. All protein, water, and ligand atoms within 18 Å of the center of the sphere were explicitly included in the simulations. Atoms close to the sphere edge were restrained to their initial coordinates and atoms beyond the sphere edge were excluded from nonbonded interactions. Asp, Glu, Lys, and Arg residues within 15 Å of the sphere center were protonated according to their most probable states at pH 7 and ionizable residues closer to the sphere edge were set to their neutral state. The protonation states of the histidines in the binding site were set by manual inspection. His278, His250, and His264 were protonated at Nδ, Nε, and both nitrogen positions, respectively. The series of 2-acetamido-benzothiazole derivatives (compounds **32–36**) were simulated with conformations of His264 and Glu169 obtained from an alternate crystal structure of the A_2A_AR^24^. In these simulations His264 was protonated at the Nδ position. The SHAKE^52^ algorithm was applied to constrain all solvent bonds and angles and the water molecules at the sphere surface were subjected to radial and polarization restraints according to the SCAAS model^51, 53^. A nonbonded cutoff of 10 Å was used for all atoms except the ligand, for which no cutoff was applied. Long-range electrostatic interactions were treated with the local reaction field method^54^. The time step was set to 1 fs and nonbonded pair lists were updated every 25 steps. In the simulations of the ligands in aqueous solution, the compound was positioned in the center of the sphere and a weak harmonic restraint was applied to a central atom (*e.g*. C5 of the adenine scaffold) to prevent it from approaching the sphere edge. Clustering of the water network in the binding site was carried out based on a simulation of 8 ns with the receptor and ligand restrained to their starting coordinates, from which 8000 snapshots were extracted and processed using the algorithm of Young *et al*.^29^.

The relative binding free energy for a pair of compounds was calculated in multiple steps using MD/FEP: (*i*) The transformation of partial charges and (*ii*) combined transformation of Lennard-Jones (LJ) and parameters involving covalent bonds in several MD/FEP calculations. If multiple heavy atoms were annihilated, a separate MD/FEP calculation was carried out to remove these in a step-wise manner. A soft-core potential was introduced for the atom in a first step, followed by removal of the resulting van der Waals potential^42, 55^. The force field parameters describing angles, bonds, and improper torsions were retained for annihilated atoms whereas the torsional potential was removed in some cases to improve convergence. The total free energy was calculated as the sum of the results obtained in each step. Each MD/FEP calculation was divided into *n* intermediate states that were equilibrated separately. The potential (*U*_*m*_) defining each state was a linear combination of energy functions describing the start- (A) and endpoint (B) of the transformation

1 $${U}_{m}=(1-{\lambda }_{m}){U}_{A}+{\lambda }_{m}{U}_{B}$$

where λ_m_ varies from 0 to 1. The FEP calculations involving partial charges were performed using 11 states whereas the number of λ values used to transform LJ and bonded parameters varied from 40 to 122 steps. Each receptor-ligand complex was equilibrated for 475 ps at each λ value. In this simulation, harmonic restraints on the protein and ligand atoms were released in several steps and the temperature was gradually increased to 310 K. The equilibration step was followed by 250 ps of unrestrained simulation, from which potential energies were extracted. The same transformation was carried out in a spherical water droplet. In this case, the system was equilibrated for 350 ps, followed by 100 ps of unrestrained simulation. The free energy difference between states *A* and *B* was calculated by summing up the free energy differences of the *n* intermediate states using

2 $${\rm{\Delta }}{G}_{A\to B}^{FEP}=-kT\,\sum _{m=1}^{n-1}\,\mathrm{ln}\,{\langle {e}^{-({U}_{m+1}-{U}_{m})/kT}\rangle }_{m}$$

where 〈…〉_*m*_ represents an ensemble average on the potential *U*_*m*_, which is calculated from the MD simulations^13^. Three replicates, which were initiated from different starting velocities for the atoms in the system, were performed for each state and these were exponentially averaged in calculations of the free energy. The uncertainty of a transformation was quantified as the difference in free energy obtained by applying the FEP formula in the forward and reverse direction and was optimized by increasing the number of λ values or simulation length until convergence was obtained. The uncertainty of a calculated relative binding free energy was estimated as the standard error of the mean of three independent trajectories.

The enthalpy (ΔΔH_bind_) and entropy (ΔΔS_bind_) contribution to the relative binding free energy were calculated from a relationship analogous to the van’t Hoff equation^28^. Each FEP transformation was carried out at 5 K intervals between 270 and 330 K to calculate the temperature dependence of the relative binding free energy (ΔΔG_bind_). The entropy and enthalpy components were then calculated from the slo﻿pe and ﻿interc﻿ept of the relation between ΔΔG_bind_/T and 1/T:

3 $$\frac{{\rm{\Delta }}{\rm{\Delta }}{G}_{bind}}{T}=\frac{1}{T}{\rm{\Delta }}{\rm{\Delta }}{H}_{bind}-{\rm{\Delta }}{\rm{\Delta }}{S}_{bind}$$

### Molecular docking and ALogP calculations

The docking calculations were carried out with GLIDE^25^ (version 6.0, Schrödinger, LLC, New York, NY, 2017) using the standard precision (SP) protocol and default settings. The same A_2A_AR crystal structure (PDB code 4EIY^17^) and protonation states for the ionizable residues as in the MD simulations were used. Prior to docking, all non-protein atoms (*e.g*. water molecules) were removed and the binding site was defined based on the co-crystallized ligand. ALogP values were calculated using Maestro (Release 2017–1: Maestro, Schrödinger, LLC, New York, NY, 2017).

### Torsion scans

Potential energy profiles for the ethyl and methoxy substituents of compounds **21** and **30** were calculated using Gaussian09^56^. The torsional scans were performed using B3LYP/DFT with the 6-311++G(d,p) basis set^57–60^ and a full geometry optimization was carried out at each point. The corresponding force field potential energies were calculated based on the geometries obtained from the DFT optimization.

### Radioligand binding and functional assays

Radioligand binding assays for compounds **25**–**36** were performed as previously described^61^ using membrane preparations from Chinese hamster ovary (CHO) or human embryonic kidney (HEK)293 cells stably expressing the human A_1_, A_2A_ or A_3_AR. The following radioligands: antagonist [^3^H]DPCPX (0.5 nM); antagonist [^3^H]ZM241385 (1.0 nM); and agonist [^125^I]AB-MECA (0.2 nM) were used for A_1_, A_2A_, and A_3_ARs, respectively. Binding parameters were calculated using Prism 6 software (GraphPAD, San Diego, CA, USA). IC_50_ values obtained from competition curves were converted to K_i_ values using the Cheng-Prusoff equation. Data were expressed as mean ± standard error. The radioligand binding assays for compounds **18**, **20**, **21**, and **23** were performed as previously described^23^. Each K_i_ value was determined from at least three independent experiments.

Four adenine derivatives (compounds **5**, **19**, **22**, and **23**) were evaluated in functional assays. CHO cells, stably transfected with the human A_2A_AR and transiently with firefly luciferase biosensor, were grown adherently and maintained in Dulbecco’s Modified Eagles Medium with nutrient mixture F12 (DMEM/F12 with phenol red), supplemented with 10% FBS (Fetal Bovine Serum), 100 U/mL penicillin, 100 µg/mL streptomycin, 2.5µg/mL amphotericin, 1 mM Sodium pyruvate, and 0.1 mg/mL Geneticin (G418) at 37 °C, and aerated with 5% CO_2_: 95% O_2_. Cells were harvested in CO_2_ independent media and counted in a Neobauer chamber. The desiderate cell number was incubated in equilibration medium containing a 3% v/v GloSensor cAMP reagent stock solution, 10% FBS and 87% CO_2_ independent medium. After 2 hours of incubation cells were dispensed in wells of 384 well plate and when a steady-state basal signal was obtained, different concentrations of antagonists were added. After 10 min, 1 µM of 5′-*N*-ethylcarboxamidoadenosine (NECA), the reference agonist, was injected. Responses were expressed as percentage of the Maximal Relative Luminescence Units (RLU). Inhibition-response curves were fitted by a non-linear regression using a Prism 4.0 program (GraphPad Software, San Diego, CA, USA).

## Electronic supplementary material

Supplementary Information
