# Supplementary material for: Fragment optimization for GPCRs by molecular dynamics free energy calculations: Probing druggable subpockets of the A2A adenosine receptor binding site
Source: Sci Rep. 2017 Jul 25;7:6398. doi: 10.1038/s41598-017-04905-0 (PMC5526870; doi:10.1038/s41598-017-04905-0)
Supplement: Supplementary file 1 — Supplementary Information [file 41598_2017_4905_MOESM1_ESM.pdf]

Supplementary information:

Fragment optimization for GPCRs by molecular dynamics free energy calculations: Probing druggable subpockets of the A_{2A} adenosine receptor binding site

Pierre Matricon[1,§], Anirudh Ranganathan[2,§], Eugene Warnick[3], Zhan-Guo Gao[3], Axel Rudling[2], Catia Lambertucci[4], Gabriella Marucci[4], Aitakin Ezzati[2], Mariama Jaiteh[1], Diego Dal Ben[4], Kenneth A. Jacobson[3], and Jens Carlsson[1,*]

[1]Science for Life Laboratory, Department of Cell and Molecular Biology, Uppsala University, SE-75124 Uppsala, Sweden.
[2]Science for Life Laboratory, Department of Biochemistry and Biophysics, Stockholm University, SE-106 91 Stockholm, Sweden.
[3]Molecular Recognition Section, Laboratory of Bioorganic Chemistry, National Institute of Diabetes and Digestive and Kidney Diseases, National Institutes of Health, Bethesda, Maryland 20892, United States.
[4]Scuola di Scienze del Farmaco e dei Prodotti della Salute, Università degli Studi di Camerino, Via S. Agostino 1, 62032 Camerino (MC), Italy.

[§] These authors contributed equally to this work.
* Corresponding author

Supplementary Table S1. Binding data for compounds **1-23**.

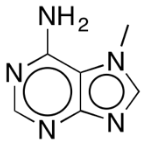

1

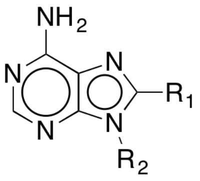

2-20

Compounds	R_1	R_2	K_i (μM)
**1**	-	-	>100[a]
**2**	H	CH_3	6.9 (3.7-13)[a]
**3**	Br	CH_3	0.12 (0.076-0.20)[a]
**4**	Br	H	3.2 (1.3-7.9)[a]
**5**	Br	CH_3CH_2	0.052 (0.024-0.11)[a]
**6**	Br	$HOCH_2CH_2$	0.62 (0.54-0.71)[a]
**7**	Br	$CH_3CH_2CH_2$	0.30 (0.26-0.35)[a]
**8**	Br	$(CH_3)_2CHCH_2$	6.0 (3.7-9.8)[a]
**9**	H	CH_3CH_2	2.2 (1.4-3.5)[a]
**10**	H	$HOCH_2CH_2$	11 (6.5-18)[a]
**11**	H	$(CH_3)_2CHCH_2$	>100[a]
**12**	H	$HOCH_2CH_2CH_2$	3.9 (3.4-4.5)[a]
**13**	H	$CH_3CH_2CH_2$	9.6 (5.8-16)[a]
**14**	Br	cC_5H_9	1.9 (1.6-2.3)[a]
**15**	H	cC_5H_9	1.8 (0.68-4.6)[a]
**16**	Br	$CH_2CHCH_2CH_2$	1.6 (1.4-1.9)[a]
**17**	H	$CH_2CHCH_2CH_2$	8.5 (5.0-15)[a]
**18**	CH_3	CH_3CH_2	0.218 ± 0.061[c]
**19**	furyl	CH_3CH_2	0.004 (0.003-0.005)[b]
**20**	HO	CH_3CH_2	1.144 ± 0.290[c]
**21**	CH_3O	CH_3CH_2	0.027 ± 0.000[c]
**22**	CH_3CH_2O	CH_3CH_2	0.046 (0.024-0.091)[b]
**23**	$(CH_3)_2CHO$	CH_3CH_2	>100[b] / 0.095 ± 0.049[c]

[a]K_i value from Lambertucci *et al.*[1]
[b]K_i value from Volpini *et al.*[2]
[c]Displacement of specific [^{3}H]NECA binding at human A_{2A}AR expressed in CHO cells measured in this work. Data is expressed as geometric means with 95% confidence limits (n=3-6).

Supplementary Table S2. Experimental data for compounds **25-36**. Radioligand binding assays were performed using membranes of mammalian cells overexpressing one AR subtype.

Compounds	K_i (μM) or % inhibition at 300 μM (n=3)[a]		
	A_{2A}	A_1	A_3
**25**	34 ± 1%	27 ± 4%	39 ± 2%
**26**	78.5 ± 2.5	44 ± 4%	50 ± 4%
**27**	20.0 ± 1.5	97.3 ± 3.5	119 ± 42
**28**	17 ± 4%	22 ± 6%	153 ± 31
**29**	31 ± 3%	38 ± 6%	44 ± 4%
**30**	49 ± 1%	97.2 ± 22.5	110 ± 17
**31**	223 ± 17	50.1 ± 22.6	102 ± 32
**32**	79.1 ± 15.2	11.2 ± 2.4	0.81 ± 0.17
**33**	10.7 ± 2.3	17 ± 1.2	2.3 ± 0.5
**34**	11.6 ± 1.2	8.4 ± 0.9	1.1 ± 0.2
**35**	48.6 ± 2.6	17.8 ± 1.7	2.9 ± 0.4
**36**	1.8 ± 0.05	7.8 ± 0.5	1.0 ± 0.2

[a]Data are expressed as mean ± standard error resulting from three independent experiments.

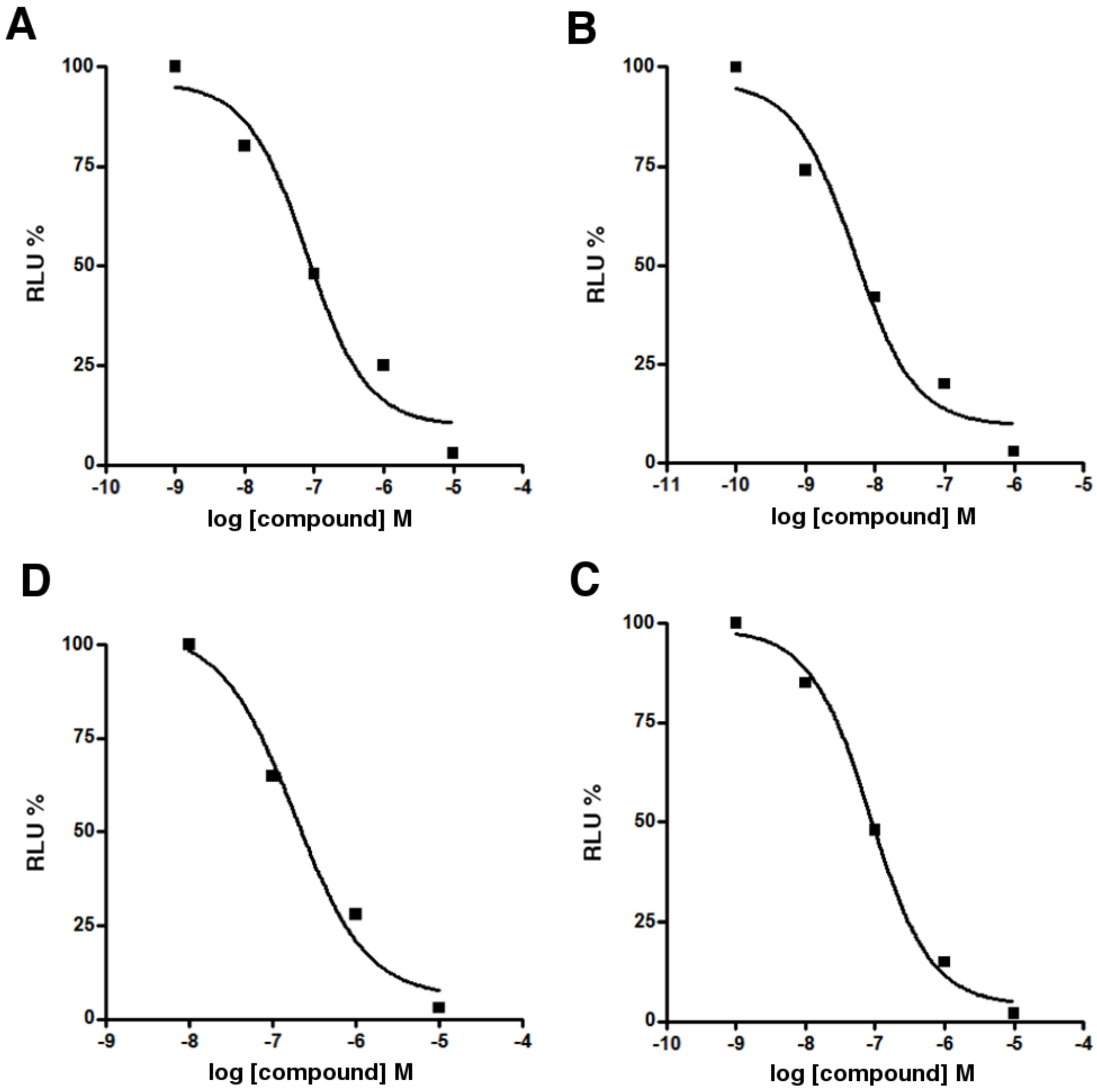

Supplementary Figure S1. Functional assay measuring the inhibition of $A_{2A}AR$ mediated cAMP production by compounds **5**, **19**, **22**, and **23** (A, B, C, and D, respectively). All the compounds close to completely inhibit the agonist effect of 1 µM NECA (a reference $A_{2A}AR$ agonist), as expected for competitive antagonism.

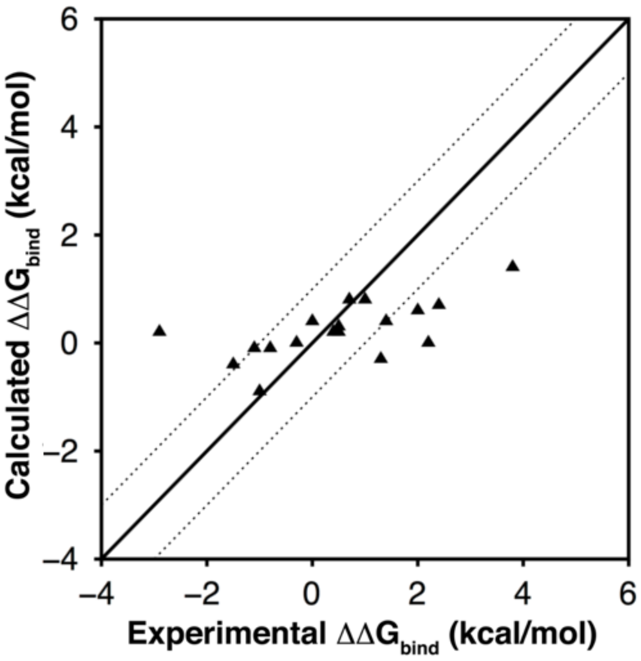

Supplementary Figure S2. Comparison of calculated (GLIDE-SP) and experimental relative binding free energies for 18 adenine-derived compound pairs. The solid line represents prefect agreement between calculated and experimental data whereas the dotted lines represent an absolute deviation of 1 kcal/mol.

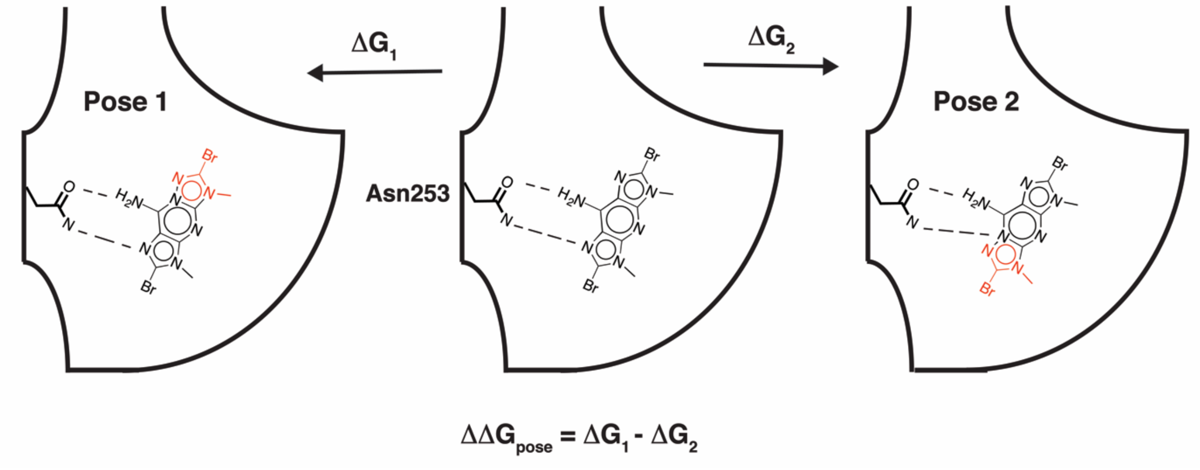

Supplementary Figure S3. Evaluation of the relative binding free energy for two alternative binding poses for compound **3**. MD/FEP transformations from the intermediate compound (**24**) to each pose were performed. The atoms that are annihilated from compound **24** in each pose are shown in red.

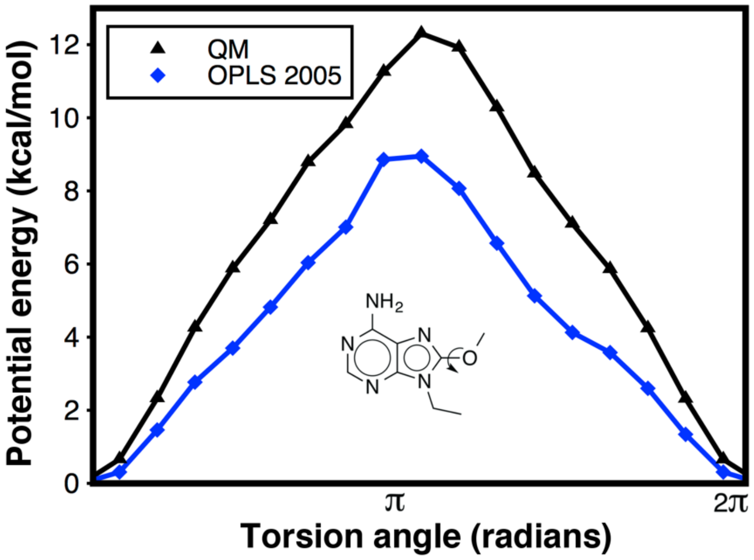

Supplementary Figure S4. Potential energy curve for the indicated torsion of compound **21** calculated from using OPLS_2005 and DFT.

Supplemental references

1. Lambertucci, C. *et al.* 8-Bromo-9-alkyl adenine derivatives as tools for developing new adenosine A_{2A} and A_{2B} receptors ligands. *Bioorg Med Chem* **17**, 2812-2822 (2009).

2. Volpini, R. *et al.* Adenosine A_{2A} Receptor Antagonists: New 8-Substituted 9-Ethyladenines as Tools for in vivo Rat Models of Parkinson's Disease. *Chemmedchem* **4**, 1010-1019 (2009).
